# Bim Links ER Stress and Apoptosis in Cells Expressing Mutant SOD1 Associated with Amyotrophic Lateral Sclerosis

**DOI:** 10.1371/journal.pone.0035413

**Published:** 2012-04-16

**Authors:** Kai Y. Soo, Julie D. Atkin, Manal Farg, Adam K. Walker, Malcolm K. Horne, Phillip Nagley

**Affiliations:** 1 Department of Biochemistry and Molecular Biology, Monash University, Clayton, Victoria, Australia; 2 Department of Biochemistry, La Trobe Institute of Molecular Science, La Trobe University, Bundoora, Victoria, Australia; 3 Florey Neuroscience Institutes, University of Melbourne, Parkville, Victoria, Australia; 4 Centre for Neuroscience, University of Melbourne, Parkville, Victoria, Australia; 5 Department of Neurology, St Vincent's Hospital, Fitzroy, Victoria, Australia; National Institutes of Health, United States of America

## Abstract

Endoplasmic reticulum (ER) stress is an important pathway to cell death in amyotrophic lateral sclerosis (ALS). We previously demonstrated that ER stress is linked to neurotoxicity associated with formation of inclusions of mutant Cu,Zn-superoxide dismutase 1 (SOD1). Cells bearing mutant inclusions undergo mitochondrial apoptotic signalling. Here, we demonstrate that the BH3-only protein, Bim, is a direct link between ER stress and mitochondrial apoptosis. In the murine neuroblastoma cell line, Neuro2a, bearing mutant SOD1 inclusions, indicators of both ER stress and apoptosis are expressed. Bim knockdown by siRNA significantly reduced nuclear apoptotic features in these inclusion-bearing cells (but did not affect the proportion of cells overall that bear inclusions). Further, both Bax recruitment to mitochondria and cytochrome *c* redistribution were also decreased under Bim-depletion conditions. However, upregulation of CHOP, a marker of ER stress, was not reduced by Bim knockdown. Significantly, knockdown of CHOP by siRNA reduced the extent of apoptosis in cells bearing mutant SOD1 inclusions. These sequential links between ER stress, CHOP upregulation, and Bim activation of mitochondrial apoptotic signalling indicate a clear pathway to cell death mediated by mutant SOD1.

## Introduction

Amyotrophic lateral sclerosis (ALS) represents 85% of all motor neuron disease in humans and is characterized by the degeneration and death of motor neurons [1], [2], [3]. In ALS, degenerating motor neurons characteristically contain proteinaceous cytoplasmic inclusions, leading to the view that ALS is a protein aggregation disorder. In transgenic mice, the appearance of mutant Cu,Zn-superoxide dismutase 1 (mSOD1) as visible cellular inclusions, derived from oligomerized mSOD1, correlates with disease progression [4], [5], suggesting a link to toxicity [6], [7]. Several theories have been proposed for the possible toxicity associated with SOD1 aggregates [8], [9], including impaired axonal transport, decreased chaperone activity, mitochondrial and proteasomal dysfunction, and the involvement of endoplasmic reticulum (ER) stress. ER stress is present in spinal cords of humans with sporadic ALS and in both animal and cellular models of ALS produced by over-expression of mSOD1 [10], [11], [12], [13].

We previously showed using NSC-34 cells (a motor neurone-like cell line) that activation of the mitochondrial apoptotic signalling pathway was correlated specifically with cells bearing ALS-linked mSOD1 inclusions [14]. However, it seems likely that apoptosis is not initiated directly by inclusions themselves, but rather by some upstream process activated by a toxic species (perhaps mSOD1 oligomers), as has been suggested for ALS (using animal models, transgenic mice expressing mutant SOD1 [15], [16]) and other neurodegenerative diseases [reviewed in refs [15], [16], [17], [18]. Indeed, ER stress detected by various markers including PERK and ATF6, occurs early in pathogenesis of ALS in SOD1^G93A^ transgenic mice [10]. Furthermore, in NSC-34 cells expressing SOD1 A4V (another pathogenic variant of SOD1), upregulated expression of CCAAT/-enhancer-binding protein homologous protein (CHOP) occurs, recognized as enhanced immunoreactivity of this protein in the nucleus [19]. Such upregulation of CHOP is thought to reflect a critical stage in the cellular response to ER stress, as a transitional phase to cellular demise [20]. Note that ER stress in NSC-34 cells and Neuro2a cells (a mouse neuroblastoma cell line) most often occurs prior to the formation of visible inclusions [12], [19].

There is accumulating evidence demonstrating that ER stress may activate the mitochondrial apoptotic pathway that results in the release of cytochrome *c* (cyt c) from the mitochondria [21], [22]. Previous studies had demonstrated that proapoptotic BH3-only proteins, Bim or Puma, were required for ER stress-induced apoptosis [23], [24]. In this work, we tested the proposition that Bim provides a molecular link between the ER stress evident in model neural cells expressing mSOD1 [10], leading to mobilization of CHOP [19], and the mitochondrial apoptotic signalling that occurs in these cells [14]. Demonstration of such a link would be significant, because Bim has been implicated in the molecular processes associated with pathology and cell death in transgenic mice expressing SOD1 G93A [25]. Here we studied murine neuroblastoma Neuro2a cells subjected to Bim (or CHOP) knockdown using short interfering RNA (siRNA). Cells depleted for either of these proteins were protected against the mitochondrial apoptotic signaling that takes place in cells undergoing response to toxic effects induced by mSOD1, marked by intracellular deposition of inclusions. Moreover, ER stress was not reduced in Bim-depleted cells bearing mSOD1 inclusions. These findings constitute primary evidence that Bim is the intermediary between ER stress and mitochondrial apoptosis in cells containing potentially toxic mSOD1 proteins.

## Results

### The frequency of apoptotic nuclei is decreased in cells expressing mSOD1 inclusions after siRNA knockdown of Bim

**Figure 1:**
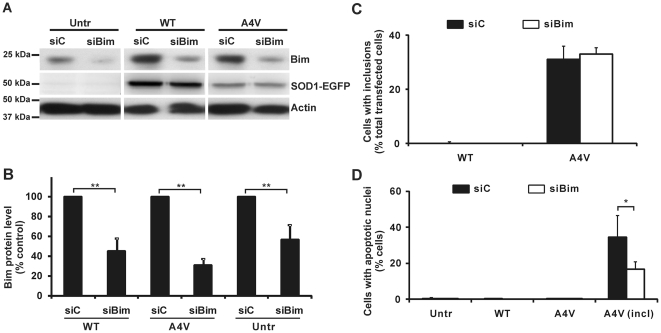
Frequencies of SOD1-inclusions and nuclear morphology changes after Bim knockdown. (A) Neuro2a cells were transfected with siRNA targeting Bim (siBim) or with non-specific control RNA (siC). After 24 h transfection of siRNA, cells were transfected with WT or SOD1 A4V-EGFP vectors for a further 48 h, or not further transfected (Untr). Cell lysates were subjected to Western immunoblotting and tested for binding of anti-Bim antibody and anti-GFP antibody. Western blot with β-actin antibody was used for control of equivalent protein loading. (B) Protein content of Bim corresponding to bands in the Western blots of panel A was quantified, first normalized to β-actin band intensity for the relevant lane, and then expressed as percentage of Bim measured in cells expressing control siC. (C) Quantification of prevalence of SOD1-inclusions. In each replicate experiment, 200 cells were scored for each population. (D) Quantified data for cells with apoptotic nuclei. For the populations containing untransfected cells (Untr) or cells expressing dispersed SOD1 (either WT or A4V), 200 cells were scored in each replicate experiment. For inclusion-positive cells (incl), more than 60 cells were scored in each of three replicate experiments. For panels B, C and D, results are expressed as mean ± SD, n = 3. * *p*<0.05; ** *p*<0.0001.

In general, transfection efficiencies of Neuro2a cells with vectors expressing WT SOD1-EGFP or SOD1 A4V-EGFP are very similar, around 50% (data not shown). However, in Neuro2a cells SOD1 A4V-EGFP is expressed at slightly reduced levels with respect to WT SOD1-EGFP, consistent with previous observations on expression of mSOD1 in Neuro2a and NSC-34 cells [12], [19]. Here, Neuro2a cells were transfected with either control or Bim siRNA for 24 h and further transfected with WT SOD1 or SOD1 A4V-EGFP vector for another 48 h. Lysates were prepared and expression of Bim protein was examined by Western immunoblotting (Figure 1A). After Bim siRNA transfection, the cellular level of Bim protein expression in untransfected control cells (cells not transfected with SOD1-EGFP vector), or cells expressing WT SOD1, mSOD1, was reduced by at least 50% in each case, compared to cells transfected with control siRNA (Figure 1B). Such reduction in Bim expression could not be obtained with NSC-34 cells previously studied [14]. Note that SOD1-EGFP expression was consistent comparing cells transfected with either control or Bim siRNA (Figure 1A). Neuro2a cells were further studied as follows.

The frequencies of cells bearing inclusions of SOD1-EGFP were examined by confocal microscopy after Bim knockdown in Neuro2a cells expressing WT or mSOD1. As expected, inclusions were rarely observed in cells expressing WT SOD1 and treated with either Bim siRNA or control siRNA (Figure 1C). The frequency of inclusions, as a proportion of the total transfected (fluorescent) cells expressing SOD1 A4V (in the presence of control siRNA), was approximately 30% (Figure 1C). Moreover, the percentage of cells forming inclusions was very similar in cells expressing Bim siRNA and control siRNA (Figure 1C), showing that Bim depletion does not affect the formation of inclusions.

However, the induction of apoptosis associated with formation of mSOD1 inclusions was reduced in Neuro2a cells after Bim siRNA treatment. The changes in nuclear morphology monitored by DNA staining were significantly reduced in those cells bearing inclusions (Figure 1D). Thus, as previously reported [14], 35% of cells bearing SOD1 A4V inclusions contained apoptotic nuclei when Bim signalling is intact (i.e. in the absence of Bim siRNA, Figure 1D), consistent with that found with NSC-34 cells. But when Bim siRNA is present, the proportion of mSOD1 inclusion-bearing cells with apoptotic nuclei was significantly decreased (*p*<0.05) to approximately 20% (Figure 1D).

### Translocation of CHOP to nucleus in cells bearing mSOD1 inclusions is not affected by the knockdown of Bim by siRNA

**Figure 2:**
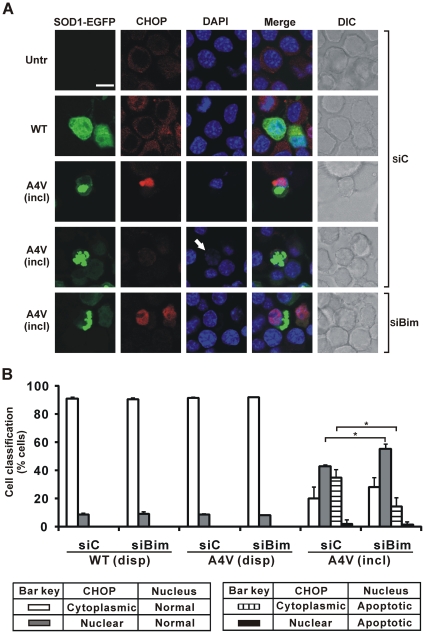
Impact on Bim depletion on mobilization of CHOP to nucleus in cells. Cells were initially transfected with Bim siRNA (siBim) or non-specific control RNA (siC) as indicated, and secondarily transfected with different forms of SOD1-EGFP or not (Untr). (A) First column shows EGFP fluorescence in green channel. Cells were fixed and tested for immunoreactivity with antibodies recognizing CHOP (second column), and also stained with DAPI (third column). Merge (fourth column) indicates overlays of the confocal images of EGFP fluorescence, CHOP and DAPI. DIC images are shown in fifth column. Scale bar, 10 µm, applies to all fields. For cells transfected with mSOD1 A4V, fields displaying inclusion-positive cells (incl) are shown. White arrow indicates apoptotic nucleus; in this case DAPI staining is very weak, characteristic of many apoptotic nuclei in cells bearing inclusions, as reported elsewhere [14]. (B) Quantified data for cells displaying CHOP mobilization to nucleus and/or apoptotic nuclei. Cells were classified as having CHOP either predominantly in cytoplasm or in nucleus. Numbers of cells scored for populations with dispersed SOD1 (disp) or those with inclusion-positive cells (incl), and expression of results are exactly as for Figure 1. * *p*<0.0001.

To test whether Bim knockdown modulates ER stress in cells bearing mSOD1 inclusions, relocation of CHOP to the nucleus concomitant with its elevated expression [19] was examined in Neuro2a cells in which Bim was depleted. Cells were transfected with WT or SOD1 A4V-EGFP vector after exposure to control siRNA for 24 h, and immunocytochemistry was performed to examine cellular distribution of CHOP. Typically, immunoreactivity to CHOP is relatively weak, localized in the cytoplasm and excluded from the nucleus in untransfected Neuro2a cells and those expressing WT SOD1: nuclear morphology is also normal (Figure 2A, first and second rows, respectively). In contrast, CHOP immunoreactivity is enhanced and concentrated in the nuclear region of cells bearing mSOD1 inclusions (Figure 2, third row), indicative of upregulation of CHOP and its efficient targeting to the nucleus. In some instances, cells bearing mSOD1 inclusions possessed apoptotic nuclei in which CHOP was barely detected (Figure 2, fourth row). When Bim is depleted by siRNA treatment, in cells bearing inclusions there is strong immunoreactive CHOP, localized in nuclei with normal morphology (Figure 2, last row).

**Figure 3:**
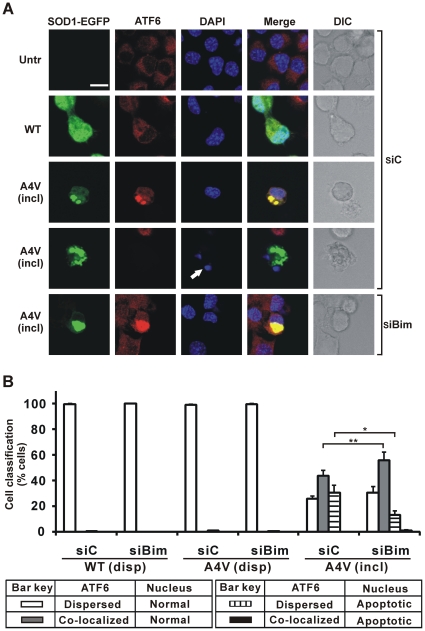
Impact of Bim depletion on co-localization of ATF6 and mSOD1 inclusions in cells. Cells were initially transfected with Bim siRNA (siBim) or non-specific control RNA (siC) as indicated, and secondarily transfected with different forms of SOD1-EGFP or not (Untr). (A) First column shows EGFP fluorescence in green channel. Cells were fixed and tested for immunoreactivity with antibodies recognizing ATF6 (second column), and also stained with DAPI (third column). Merge (fourth column) indicates overlays of the confocal images of EGFP fluorescence, ATF6 and DAPI. DIC images are shown in fifth column. Scale bar, 10 µm, applies to all fields. For cells transfected with mSOD1 A4V, fields displaying inclusion-positive cells (incl) are shown. White arrow indicates apoptotic nucleus, in this case highly condensed and fragmented revealed by DAPI staining. (B) Quantified data for cells displaying co-localization of ATF6 and mSOD1 inclusions and/or apoptotic nuclei. Numbers of cells scored for populations with dispersed SOD1 (disp) or those with inclusion-positive cells (incl), and expression of results are exactly as for Figure 1. * *p*<0.001; ** *p*<0.0001.

Applying the quantification of morphology illustrated in Figure 2A, approximately 45% of cells bearing mSOD1 inclusions but not depleted for Bim have nuclear CHOP but normal nuclei and 35% have apoptotic nuclei but barely detectable nuclear CHOP. However, Bim knockdown in cells bearing mSOD1 inclusions resulted in a significantly increased proportion of cells (60%) with nuclear CHOP and normal nuclei, and correspondingly fewer (15%) cells whose nuclei were apoptotic but without significantly detectable nuclear CHOP (Figure 2B). When cells were free of inclusions, with or without Bim depletion, the frequency of nucleus-localized CHOP (overwhelmingly with normal nuclei) was at the background level of about 10% (Figure 2B). Overall, these data indicate that knockdown of Bim in cells bearing mSOD1 inclusions strongly decreased apoptosis (by about half, from 37% to 17%) but the frequency with which CHOP relocates to the nucleus marginally increased from 45% to 58%. Therefore, ER stress, as measured by CHOP immunoreactivity in the nucleus, occurs upstream of Bim action. This conclusion was strengthened by demonstrating that relocation of ATF6 from ER to nucleus (another marker of ER stress) also slightly increased after Bim depletion (from 44% to 57%) in cells bearing inclusions of SOD1 A4V, under conditions where apoptotic nuclei decreased from 31% to 14% (Figure 3). Note that in these studies ATF6 apparently co-localized with inclusions, unlike previous reports [12], but this does not impact on the essential conclusion that Bim depletion has no significant effect on ER stress signalling in Neuro2a cells.

### The frequency of Bax recruitment to mitochondria is decreased in cells bearing mSOD1 inclusions after siRNA knockdown of Bim

**Figure 4:**
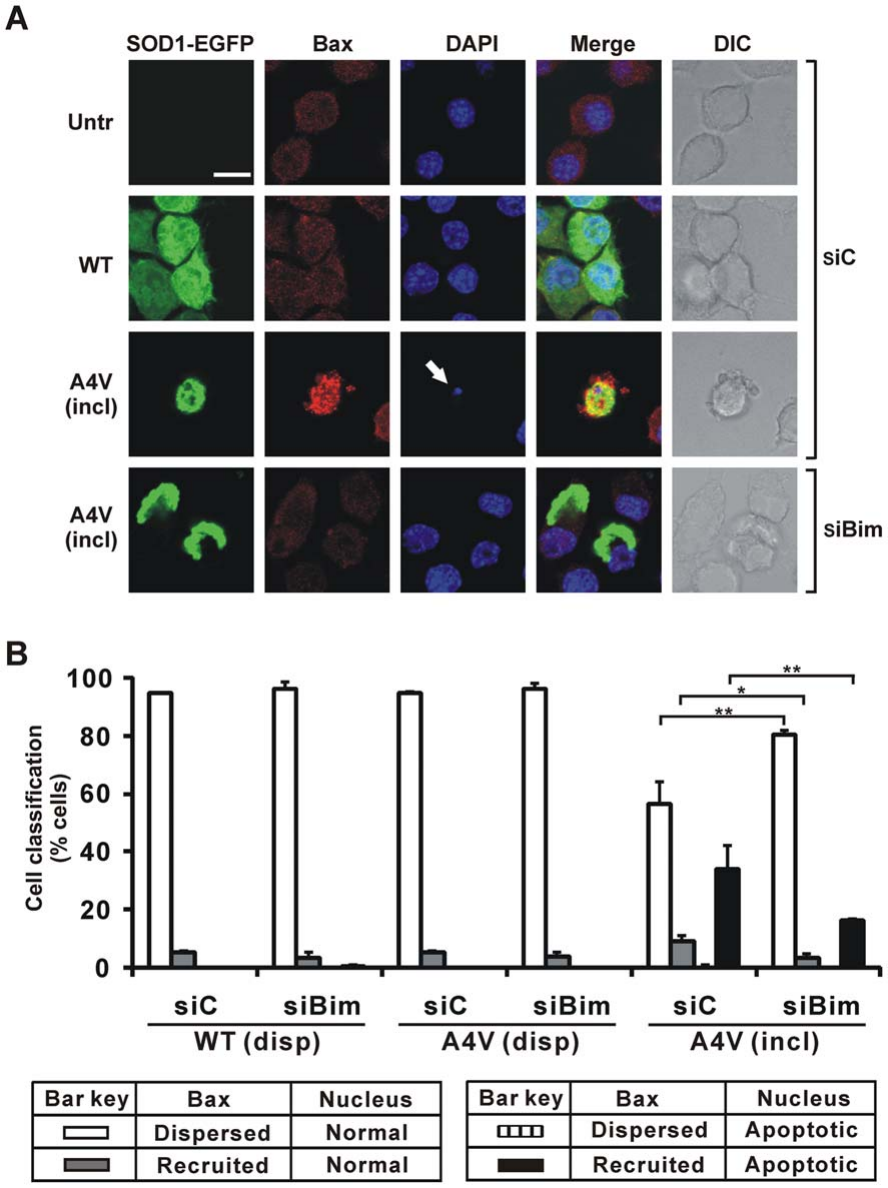
Impact of Bim depletion on recruitment of Bax to mitochondria in cells. Cells were initially transfected with Bim siRNA (siBim) or non-specific control RNA (siC) as indicated, and secondarily transfected with different forms of SOD1-EGFP or not (Untr). (A) First column shows EGFP fluorescence in green channel. Cells were fixed and tested for immunoreactivity with antibodies recognizing Bax (second column), and also stained with DAPI (third column). Merge (fourth column) indicates overlays of the confocal images of EGFP fluorescence, Bax and DAPI. DIC images are shown in fifth column. Scale bar, 10 µm, applies to all fields. For cells transfected with mSOD1 A4V, fields displaying inclusion-positive cells (incl) are shown. White arrow indicates apoptotic nucleus, with little DAPI staining (cf. Figure 2A). (B) Quantified data for cells displaying Bax recruitment and/or apoptotic nuclei. Numbers of cells scored for populations with dispersed SOD1 (disp) or those with inclusion-positive cells (incl), and expression of results are exactly as for Figure 1. * *p*<0.001; ** *p*<0.0001.

It was previously observed in NSC-34 cells bearing mSOD1 inclusions (induced by transfecting cells with SOD1 A4V-EGFP vector) that Bax recruitment to mitochondria was the earliest indicator of mitochondrial apoptotic signalling [14]. In control Neuro2a cells, either untransfected or expressing WT SOD1-EGFP, inactive Bax is typically localized to the cytosol, seen as diffuse immunoreactivity, and the nuclear morphology of these cells is normal (Figure 4A, top two rows). However, when Neuro2a cells were transfected to generate mSOD1 inclusions, Bax was recruited from the cytosol and apoptosis occurred as determined by the presence of apoptotic nuclei (Figure 4A, third row). When Bim was depleted by specific siRNA, Bax remained cytosolic and nuclear morphology was not affected in cells bearing mSOD1 inclusions, (Figure 4A, last row).

Quantification of cells shows that nuclei are apoptotic and Bax was recruited in 35% of cells bearing mSOD1 inclusions without Bim knockdown (Figure 4B). In contrast, when Bim was knocked down only 18% of inclusion-bearing cells recruited Bax and had apoptotic nuclei (Figure 4B). Overall Bax recruitment was reduced from 45% to 18%. In general, only 5% of cells without inclusions, with or without Bim depletion, recruited Bax to mitochondria, but none had apoptotic nuclei (Figure 4B). These results indicate that the formation of mSOD1 inclusions is associated with Bax recruitment in Neuro2a cells and that Bim protects against apoptosis upstream of Bax recruitment to mitochondria.

### The frequency of cyt c redistribution from mitochondria is decreased in cells bearing mSOD1 inclusions after siRNA knockdown of Bim

**Figure 5:**
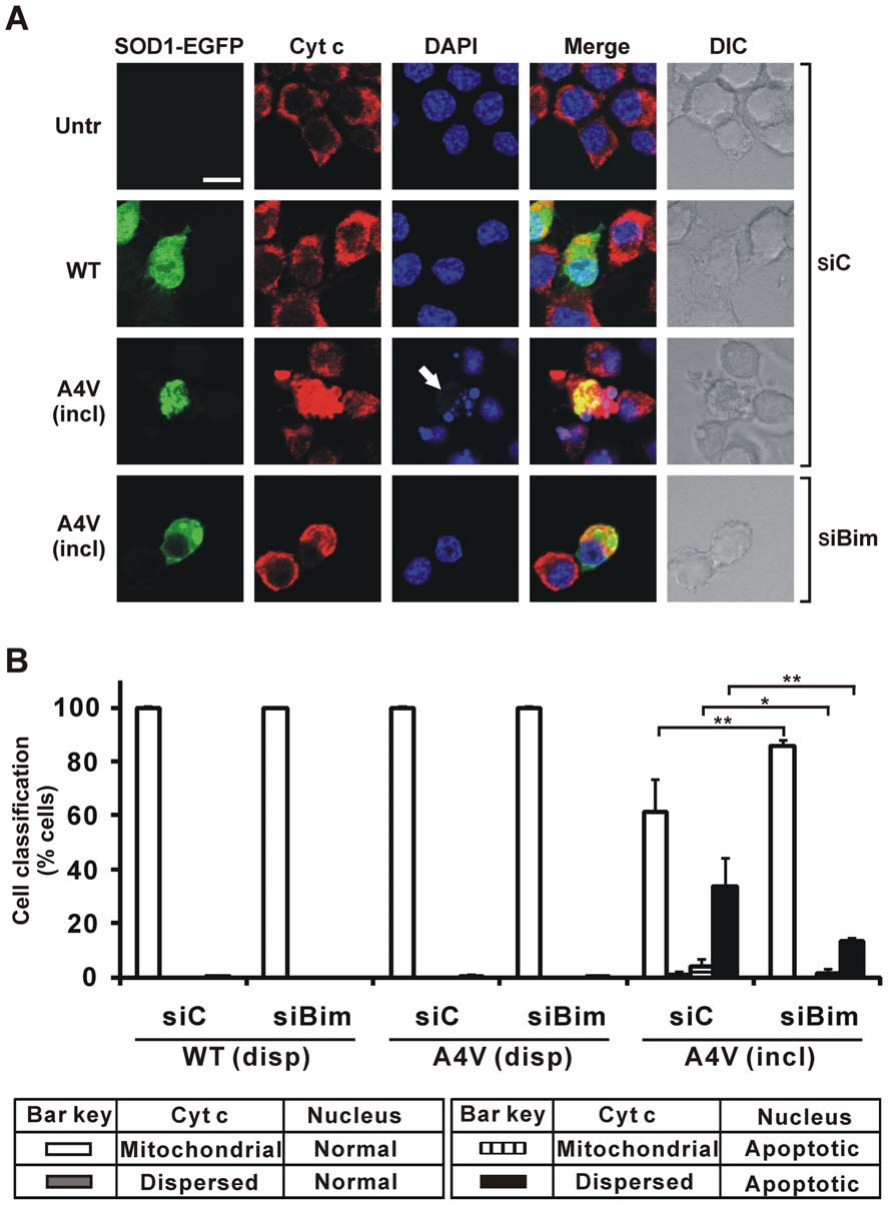
Impact of Bim depletion on redistribution of cyt c from mitochondria in cells. Cells were initially transfected with Bim siRNA (siBim) or non-specific control RNA (siC) as indicated, and secondarily transfected with different forms of SOD1-EGFP or not (Untr). (A) First column shows EGFP fluorescence in green channel. Cells were fixed and tested for immunoreactivity with antibodies recognizing cyt c (second column), and also stained with DAPI (third column). Merge (fourth column) indicates overlays of the confocal images of EGFP fluorescence, cyt c and DAPI. DIC images are shown in fifth column. Scale bar, 10 µm, applies to all fields. For cells transfected with mSOD1 A4V, fields displaying inclusion-positive cells (incl) are shown. White arrows indicates apoptotic nucleus, in this case highly condensed and fragmented revealed by DAPI staining. (B) Quantified data for cells displaying cyt c redistribution and/or apoptotic nuclei. Numbers of cells scored for populations with dispersed SOD1 (disp) or those with inclusion-positive cells (incl), and expression of results are exactly as for Figure 1. * *p*<0.001; ** *p*<0.0001.

Redistribution of cyt c from mitochondria to the cytosol was monitored in Neuro2a cells with or without Bim depletion. In untransfected control cells and also in cells expressing dispersed WT SOD1, cyt c immunoreactivity was punctuate, reflecting itsmitochondrial localization, and negligible within the nuclear region (Figure 5A). In contrast, in many cells bearing mSOD1 A4V inclusions, cyt c immunoreactivity was distributed across the whole cell area, with some coincident with apoptotic nucleus (Figure 5A, third row). However, in Bim-depleted cells bearing mSOD1 inclusions, cyt c remained in the mitochondria and few,if any, apoptotic nuclei were observed (Figure 5A, last row).

Quantification of the cell populations revealed that approximately 35% of cells bearing mSOD1 inclusions, but not depleted for Bim, had both apoptotic nuclei and cyt c redistribution (Figure 5B). However, in cells bearing mSOD1 inclusions, under conditions of Bim knockdown, the proportion of those expressing apoptotic features was reduced to 10%. At the same time, the proportion of cells displaying mitochondria-localized cyt c with normal nuclear morphology rose to 90%, compared to such cells (65%) in populations exposed to control siRNA (Figure 5B). All other cells without inclusions had normal nuclear morphology and little detectable cyt c redistribution with or without Bim knockdown (Figure 5B). Therefore, these results confirm that knockdown of Bim rescues cells from apoptosis via the mitochondrial apoptotic pathway.

### Knockdown of CHOP decreases apoptosis in cells bearing mSOD1 inclusions

**Figure 6:**
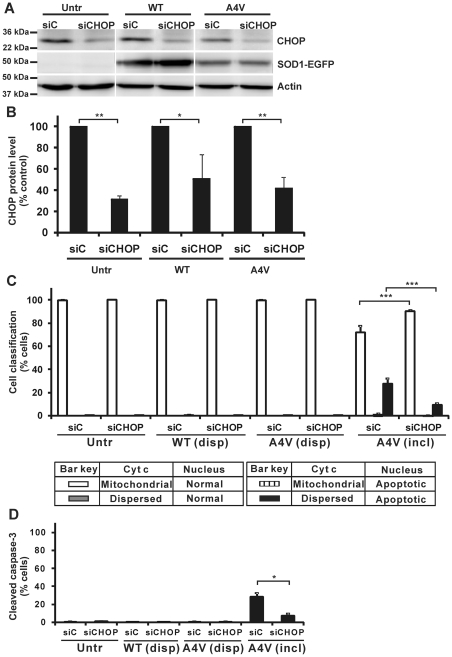
Impact of CHOP depletion on cyt c redistribution from mitochondria and caspase-3 cleavage in cells. (A) Neuro2a cells were transfected with siRNA targeting CHOP (siCHOP) or with non-specific control RNA (siC). After 24 h transfection of siRNA, cells were transfected with WT or SOD1 A4V-EGFP vectors for a further 48 h, or not further transfected (Untr). Cell lysates were subjected to Western immunoblotting and tested for binding of anti-CHOP antibody and anti-GFP antibody. Western blot with β-actin antibody was used for control of equivalent protein loading. (B) Protein content of CHOP corresponding to bands in the Western blots of panel A was quantified, first normalized to β-actin band intensity for the relevant lane, and then expressed as percentage of CHOP measured in cells expressing control siC. (C) Quantified data for cells displaying cyt c redistribution and/or apoptotic nuclei. (C) Quantified data for cells displaying cleaved caspase-3. Numbers of cells scored for populations with dispersed SOD1 (disp) or those with inclusion-positive cells (incl), and expressing of results are exactly as for Figure 1. * *p*<0.05; ** *p*<0.001; *** *p*<0.0001.

To establish that CHOP is indeed a key factor in relaying the ER stress response into death signalling, CHOP depletion was carried out. Neuro2a cells were transfected with either control or CHOP siRNA and co-transfected with SOD1-EGFP vectors as described above. Lysates were prepared and expression of CHOP protein was examined by Western immunoblotting (Figure 6A). The cellular level of CHOP in the cultures after CHOP siRNA transfection was reduced to about 40 to 50% in each case, compared to cells transfected with control siRNA (Figure 6B). However, expression of neither WT SOD1-EGFP nor SOD1 A4V-EGFP was affected by knockdown of CHOP by siRNA (Figure 6A). As for Bim depletion (Figure 1C), CHOP siRNA knockdown did not affect the frequencies of cells bearing mSOD1 inclusions (data not shown). However, CHOP knockdown resulted in significant reduction in apoptotic markers in cells bearing mSOD1 inclusions (Figure 6C). Thus, cells indicating cyt c redistribution and apoptotic nucleus declined from 37% to 10%. At the same time, the proportion of cells displaying mitochondria-localized cyt c with normal nuclear morphology rose to 90%, compared to such cells (70%) in populations exposed to control siRNA (Figure 6C). All other cells without inclusions, with or without CHOP knockdown, had normal nuclear morphology and negligible cyt c redistribution (Figure 6C). Another apoptotic marker, cleavage of caspase-3 (detected by immunocytochemistry) revealed that CHOP siRNA knockdown significantly reduced cells with cleaved-caspase-3 (9%) in cells bearing mSOD1 inclusions, compared to such cells previously exposed to control siRNA (30%). Therefore, these results clearly indicate that CHOP acts upstream of mitochondrial apoptotic signalling leading to downstream caspase activation.

## Discussion

### Molecular events leading from mutant SOD1 via ER stress to apoptosis

Here, we have clearly demonstrated that the BH3-only protein, Bim, provides a key molecular link between ER stress and mitochondrial apoptosis in neuron-like cells under stress from expression of mSOD1. Thus, in Neuro2a cells bearing mSOD1 inclusions, knockdown of Bim (by siRNA) decreased mitochondrially-mediated apoptosis but not ER stress. Further, when CHOP was likewise knocked-down, apoptosis in cells bearing mSOD1 inclusions was similarly reduced. Bim depletion did not affect markers of ER stress (including CHOP), confirming that CHOP is upstream of Bim activation.

**Figure 7:**
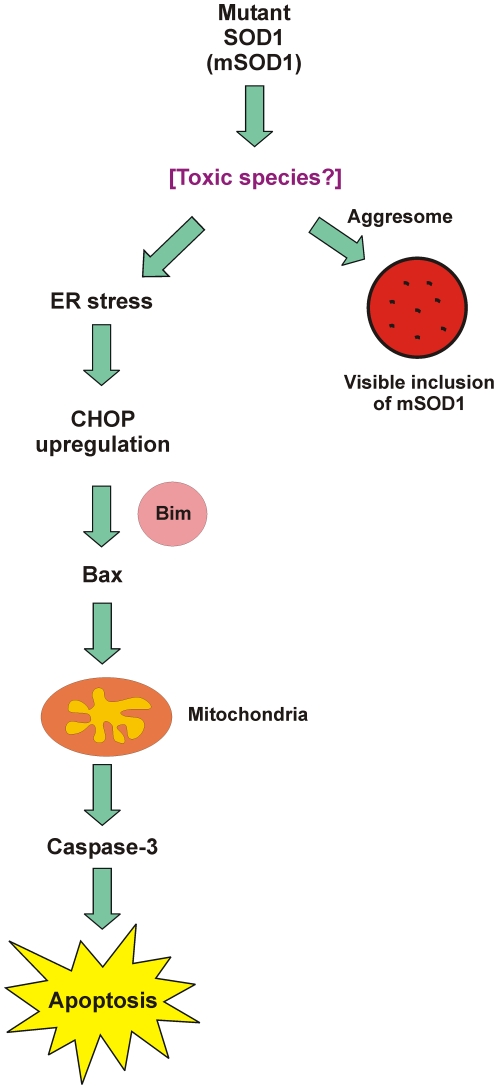
Inter-relationship between cellular responses associated with the formation of mSOD1 inclusions, ER stress and apoptosis. The primary response is thought to be elicited by the accumulation of a toxic species derived from mSOD1. One consequence is the formation of mSOD1 inclusions, via the aggresome. In parallel, ER stress is thought to be invoked if the toxic species are not destroyed by the ubiquitin-proteasome system, resulting in upregulation of CHOP. As demonstrated here the BH3-only protein, Bim, links ER stress to apoptosis. Specifically, this involves activation of apoptotic signalling pathways that sequentially include Bax recruitment to mitochondria, cyt c redistribution, caspase-3 activation and apoptosis.

A schematic outlining this molecular linkage by Bim of ER stress and apoptosis, contextualized to the possible toxic roles of mSOD1, is presented in Figure 7. The schematic encapsulates the notion that expression of mSOD1 leads to formation of some toxic species (yet to be identified) leading to proteopathic disease caused by such misfolded proteins. ER stress occurs as an early event that leads to mitochondrially-mediated apoptosis, through CHOP upregulation and subsequent Bim activation.

A direct causative effect of SOD1 inclusions on neuronal cell death is not considered likely. Rather, the formation of inclusions of mSOD1 represent a cellular default process to sequester such misfolded proteins or large aggregates that cannot be dealt with (in soluble forms) by either autophagy or the ubiquitin-proteasome system [reviewed in ref. 26]. The aggresome [27] represents an early step in the pathway leading to inclusions but is separate from that leading to ER stress, as depicted in Figure 7.

Whilst Figure 7 depicts mSOD1 as the generator of toxic species initiating the cascade of cellular disruption, it is conceivable that other proteins implicated genetically and functionally in ALS pathology (including TDP-43 and FUS/TLS) may also act to precipitate ER stress and the resultant death process mediated by Bim. It is noteworthy that all three ALS-linked proteins (SOD1, TDP-43 and FUS) have each been found in aggregated forms in pathogenic contexts [28].

Bim is essential for ER stress-induced apoptosis in a diverse range of cell types, both in cultured cells and within the whole animal. ER stress activates Bim through two novel pathways, via protein phosphatase 2A-mediated dephosphorylation, which prevents ubiquitination and proteasomal degradation, as well as by CHOP-mediated direct transcriptional induction [23]. This is consistent with the findings of this study demonstrating that CHOP immunoreactivity was not affected when Bim was knocked-down in Neuro2a cells, suggesting that CHOP is upstream of Bim [23]. CHOP-mediated activation of Bim, then leads to the activation of Bax on outer mitochondrial membrane, which results in cyt c redistribution from mitochondria and activation of the downstream caspase cascade, as set out in Figure 7.

Bim is upregulated in a familial ALS mouse model of disease during the symptomatic stage of disease [25]. Bim is activated via CHOP during ER stress [23] and the JNK and p38 kinase pathways [29]. Activation of both these pathways has been described in activated microglial cells of SOD1^G93A^ transgenic mice at the symptomatic stage of disease. Phosphorylation of Bim by the JNK pathway increases its pro-apoptotic activity, which is known to trigger neuronal death in models of ischemia-reperfusion [30]. Interestingly, Bim is sequestered to the microtubule-associated dynein motor complex when it is inactive. However when it is activated by phosphorylation, Bim is released and translocates to Bcl-2, resulting in Bax-dependent apoptosis [31]. Mutant SOD1 disrupts the dynein/dynactin interaction leading to impaired axonal transport in motor neurons, and dysfunction of dynein leads to motor degeneration [32], [33], [34]. Therefore, post-translational modifications of Bim may play a role in motor neuron loss in familial ALS, but we have not yet addressed such issues in our cellular model system.

Puma has been shown as another BH3-only protein linking ER stress to apoptosis [24]. In this perspective, Puma was shown to be critically involved in disease progression in SOD1^G93A^ transgenic mice [35] and deletion of Puma was found to protect cells from ER stress-induced cell death [36]. Here we emphasize the role of Bim in linking ER stress to apoptosis in cells expressing mSOD1. CHOP is clearly implicated upstream of Bim but the detailed mechanism remains to be defined.

## Materials and Methods

### Cell culture and transfection

Neuroblastoma Neuro2a cells (ATCC number: CCL-131) were maintained in DMEM supplemented with 2 mM L-glutamine, 10 mM HEPES buffer, and 10% (v/v) fetal calf serum. Cells were maintained in a humidified 37°C incubator with 5% CO_2_. WT SOD1 and mSOD1 (A4V and G85R) constructs encoding enhanced green fluorescent protein (EGFP)-tagged human SOD1 at the C-terminus were generated as previously described [37].

In order to down-regulate the expression of Bim or CHOP, cells were transfected with siRNA targeting the Bim or CHOP gene (Dharmacon, USA). Neuro2a cells were seeded a day before transfection at 5×10^4^ cells/well of a 12-well plate in cDMEM and incubated under normal growth conditions (37°C and 5% CO_2_). On the day of transfection, siRNA targeted to the Bim or CHOP gene was diluted at 20 nM or 100 nM, respectively, in 50 µl of OPTI-MEM I reduced-serum media (Invitrogen, USA). Lipofectamine 2000 reagent (Invitrogen, USA) (1 µl) was then diluted with 50 µl OPTI-MEM I reduced-serum media in another tube and incubated for 5 min at room temperature. After incubation, the siRNA solution was mixed with Lipofectamine 2000 reagent. This was then incubated for a further 20 min at room temperature. Meanwhile, the medium of the cells was replaced with fresh cDMEM. After incubation, the DNA-Lipofectamine 2000 reagent complexes were then added into each well in a circular drop-wise fashion to evenly distribute the complexes throughout the well. Cells were then incubated for 5 h at 37°C with 5% CO_2_. After 5 h, the medium in each well was replaced with fresh cDMEM that allowed normal growth of cells. After 24 h, the medium was replaced again with fresh cDMEM to maintain cell viability and cultures returned to the incubator. DNA was then transfected a day after siRNA transfection. After 24 h siRNA transfection, cells were transfected with WTSOD1-EGFP or SOD1 A4V-EGFP using Lipofectamine 2000. The total amount of DNA used in each well was 0.5 µg. Cells were analysed after 48 h DNA transfection, so the total siRNA transfection period was 72 h.

### Western blotting

Cells were collected and resuspended in cold cell lysis buffer (1% Triton X-100, 4 mM EGTA, 15 mM MgSO_4_ 25 mM glycyglycine) and incubated on ice for 5 min. Cells were centrifuged at 10, 000× g for 5 min and the cell debris discarded. Protein concentration of the cell lysates was determined using a bicinchoninic acid assay kit (Pierce, USA). Protein sample (20–30 µg) were electrophoresed through 12% SDS-polyacrylamide gels and transferred to nitrocellulose membrane. Membranes were blocked with 5% skim milk in PBS for 1 h then incubated with primary antibodies against the following proteins at 4°C for overnight: Bim (1∶1000) (Calbiochem, Darmstadt, Germany), CHOP (1∶200) (Santa Cruz, USA), GFP (1∶1000) (provided by Prof. M.T. Ryan, La Trobe University), or β-actin (1∶500) (NeoMarkers, USA). Membranes were incubated for 1 h at room temperature with secondary antibodies (1∶10,000, HRP-conjugated goat anti-mouse or goat anti-rabbit) (Pierce, USA), and were detected using ECL reagent (Pierce, USA). Quantification of blots was performed by ImageQuant TL densitometry program (GE Healthcare, Australia).

### Immunocytochemistry and confocal imaging

Immunocytochemistry and confocal imaging were carried out exactly as previously described [14]. The primary antibodies were mouse monoclonal anti-Bax antibody (clone 6A7) (1∶200) (BD Biosciences Pharmingen, USA), mouse monoclonal anti-cyt c (1∶500) (BD Biosciences Pharmingen, USA), mouse monoclonal anti-CHOP (1∶50) (Santa Cruz, USA), or rabbit polyclonal anti-ATF6 (1∶50) (Santa Cruz, USA). The secondary antibodies were Alexa 568-labelled goat anti-rabbit IgG (1∶250) (Invitrogen-Molecular Probes, USA).

### Statistical analysis

All data are expressed as the mean ± standard deviation (SD) values. The data were analysed for statistical significance by ANOVA followed by Tukey's *post-hoc* test (GraphPad Prism, USA). The differences were considered significant at *p*<0.05.
